# Prize Essays

**Published:** 1853-01

**Authors:** 


### PRIZE ESSAYS.

" The conductors of the American Journal of Dental Science, will give for the best essays on the following specified subjects, the amounts severally annexed. The first; second and fourth awards to be paid in *gold medals,* or in money, at the option of the successful competitors.

*First.*—Twenty-five dollars for the best Essay on the Causes, Varieties and Treatment of Odontalgia. This essay not to contain less than twenty-five nor more than fifty pages.

*Second.*—Fifty dollars for the best essay on the Diseases of the Gums and their Treatment, of not less than forty nor more than eighty pages.

*Third.*—A complete set of the Journal, from volume one to twelve, inclusive, handsomely bound in twelve volumes, worth sixty-six dollars, for the best essay on the Effects of Mercury upon the Teeth, Gums and Alveolar Processes. This paper to contain not less than forty nor more than seventy pages.

*Fourth.*—Seventy-five dollars for the best essay on Mechanical Dentistry. This shall include a description of the manner of obtaining an impression of the mouth, of making plaster models, metalic models and counter models. Striking up plates, arranging, fitting, antagonizing and mounting porcelain teeth, from a single tooth to an entire set—all of which to be illustrated with suitable drawings, and not to contain less than seventy nor more than one hundred and forty pages.

Each of the pages of the foregoing essays shall equal in amount of matter one of the pages of the Journal.

The essay for the first award must be received by the 20th of February; for the *second* and *third,* by the 20th of May; and for the *fourth* by the 15th of September, 1853.

Each essay must be accompanied with the name of the author in a sealed envelop or separate piece of paper. This will not be opened until after the award shall have been made.

